# Histone deacetylase inhibition protects hearing against acute ototoxicity by activating the Nf-*κ*B pathway

**DOI:** 10.1038/cddiscovery.2015.12

**Published:** 2015-07-27

**Authors:** W S Layman, D M Williams, J A Dearman, M A Sauceda, J Zuo

**Affiliations:** 1 Department of Developmental Neurobiology, St. Jude Children’s Research Hospital, 262 Danny Thomas Place, Memphis, TN 38105, USA; 2 University of Bath, Bath, UK; 3 Department of Cell and Molecular Biology, St. Jude Children’s Research Hospital, 262 Danny Thomas Place, Memphis, TN 38105, USA

## Abstract

Auditory hair cells have repeatedly been shown to be susceptible to ototoxicity from a multitude of drugs including aminoglycoside antibiotics. Here, we found that systemic HDAC inhibition using suberoylanilide hydroxamic acid (SAHA) on adult mice offers almost complete protection against hair cell loss and hearing threshold shifts from acute ototoxic insult from kanamycin potentiated with furosemide. We also found that the apparent lack of hair cell loss was completely independent of spontaneous or facilitated (ectopic *Atoh1* induction) hair cell regeneration. Rather, SAHA treatment correlated with RelA acetylation (K310) and subsequent activation of the Nf-*κ*B pro-survival pathway leading to expression of pro-survival genes such as *Cflar* (*cFLIP*) and *Bcl2l1* (*Bcl-xL*). In addition, we also detected increased expression of pro-survival genes *Cdkn1a* (*p21*) and *Hspa1a* (*Hsp70*), and decreased expression of the pro-apoptosis gene *Bcl2l11* (*Bim*). These data combined provide evidence that class I HDACs control the transcriptional activation of pro-survival pathways in response to ototoxic insult by regulating the acetylation status of transcription factors found at the crossroads of cell death and survival in the mammalian inner ear.

## Introduction

Hearing loss is the third most common health impairment and the most common occupational illness in the United States. Many things contribute to loss of hearing including aging, illness, acoustic trauma, and genetic predisposition. In addition, mammalian auditory hair cells have repeatedly been shown to be susceptible to ototoxicity from a multitude of drugs including aminoglycoside antibiotics, loop diuretics, platinum-based chemotherapy agents, and a number of nonsteroidal anti-inflammatory drugs (NSAIDS). Unlike non-mammalian vertebrates, mammals are unable to regenerate damaged auditory hair cells thereby resulting in permanent hearing loss.

Histone deacetylase (HDAC) inhibitors were originally used as anticancer agents and some are approved by the FDA for use in the treatment of specific types of cancer in humans. However, broad-spectrum and HDAC-specific inhibitors are also known to have concentration-dependent protective effects on inflammation, neurodegeneration, and oxidative stress models.^1–3^ Although HDAC inhibitors are primarily thought to modulate chromatin condensation by regulating histone acetylation and thus affect gene expression, HDAC inhibitors have also been shown to affect the posttranslational modification of some important intracellular non-histone proteins.^4,5^ However, the precise mechanism underlying the effect of HDAC inhibition in the mature mammalian inner ear remains unknown.

Here, we found that systemic HDAC inhibition using suberoylanilide hydroxamic acid (SAHA) on adult mice offers almost complete protection against hair cell loss from acute ototoxic insult (kanamycin+furosemide). Mice receiving both ototoxic insult and SAHA had little to no hair cell loss and normal hearing function. We also determined that the apparent lack of hair cell loss from ototoxic insult was completely independent of spontaneous or facilitated (Cre-mediated ectopic *Atoh1* expression) hair cell regeneration. Rather, SAHA treatment correlated with RelA acetylation (K310) and subsequent activation of the Nf-*κ*B pro-survival pathway leading to expression of pro-survival genes such as *Cflar* (*cFLIP*) and *Bcl2l1* (*Bcl-xL*). Similar to other neuroprotection studies, we also detected increased expression of pro-survival genes *Cdkn1a* (*p21*) and *Hspa1a* (*Hsp70*), and decreased expression of the pro-apoptosis gene *Bcl2l11* (*Bim*).

## Results

### Systemic SAHA treatment penetrates the adult mouse inner ear

**Figure 1:**
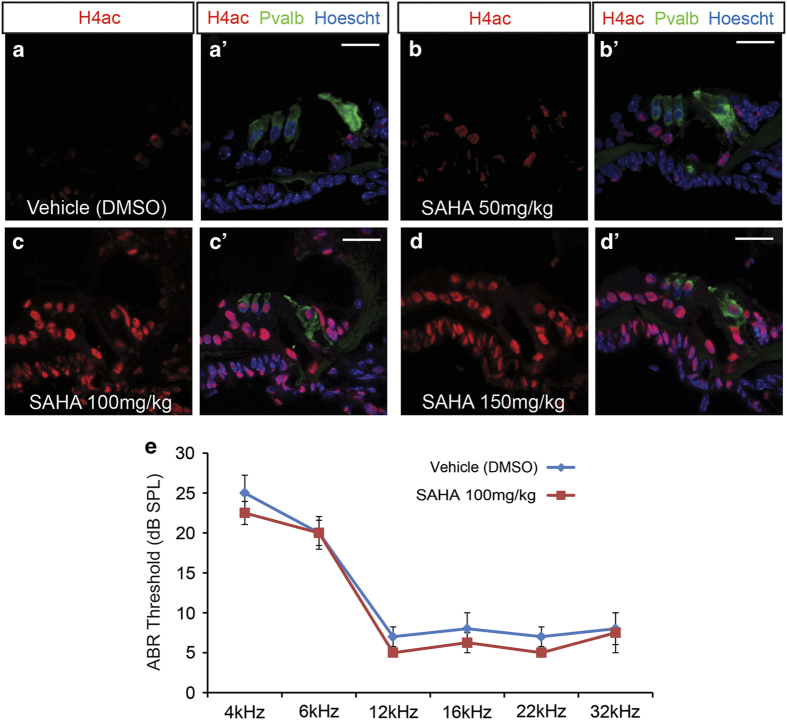
Systemically delivered SAHA penetrates the mouse inner ear. Mice were administered either vehicle (DMSO; **a** and **a’**) or SAHA at 50 mg/kg (**b** and **b’**), 100 mg/kg (**d** and **d’**), or 150 mg/kg (**d** and **d’**) by intraperitoneal injection at P28 and analyzed at P29. Immunofluorescence of organ of Corti sections was performed using antibodies against H4ac (red), Pvalb (green), and Hoechst (blue). (**a** and **a’**) The mature organ of Corti normally has very low levels of H4ac staining, similar to previous studies.^7^ (**b**–**d’**) Systemic SAHA mildly increases histone acetylation levels at 50 mg/kg, whereas both the 100 mg/kg and 150 mg/kg doses markedly increased the levels of histone H4 acetylation. (**e**) SAHA delivered systemically daily for 2 weeks did not adversely affect hearing thresholds tested by ABR. Confocal laser intensity and gain were kept consistent between samples. All representative images were taken from the middle turn of the cochlea. Scale bar is 20 *μ*m.

The adult mammalian inner ear has a remarkably stable homeostatic mechanism to maintain its functional integrity. Many regulatory mechanisms are involved in maintaining this homeostasis including ion transport, constant blood supply, and a blood–labyrinth barrier.^6^ Any disturbance in one of these mechanisms by free radicals, stress hormones, noise exposure, or aminoglycoside antibiotics may induce short- and long-term effects on cellular function.^6^ To determine whether systemic SAHA is able to both permeate the blood–labyrinth barrier and not perturb the inner ear’s homeostasis, we administered SAHA to adult C57BL/6J (B6) and FVB/NJ (FVB) wild-type mice via intraperitoneal injection using 50 mg/kg, 100 mg/kg, or 150 mg/kg SAHA dissolved in DMSO compared with vehicle-treated controls (FVB *n*=3; B6 *n*=3*;* for each dosage group). Since the mature mammalian inner ear normally has low levels of histone acetylation (Figure 1a),^7^ we used immunofluorescence to detect changes in histone H4 acetylation levels using an antibody against histone H4 pan-acetylation (H4ac). We found that 100 mg/kg SAHA caused an increase in histone H4 acetylation staining (Figure 1c), whereas 50 mg/kg had very little affect (Figure 1b). Although 150 mg/kg SAHA also dramatically increased histone H4 acetylation staining (Figure 1d), we chose to further pursue the 100 mg/kg dose for this study as higher doses of SAHA can cause cytotoxicity.^8,9^

### Systemic SAHA did not affect hearing thresholds

As systemic administration of SAHA is able to cross the blood–labyrinth barrier, we next analyzed whether repeated exposure to systemic SAHA had a detrimental impact on hearing thresholds. To determine whether mice had normal hearing thresholds, we used auditory brainstem response (ABR) to estimate hearing sensitivity and to identify whether systemic SAHA causes any neurological abnormalities of the auditory nerve and the auditory pathway up through the brainstem. Beginning at postnatal day 28 (P28), wild-type mice were injected daily for 2 weeks with either 100 mg/kg SAHA (FVB *n*=5; B6 *n*=5) or vehicle (FVB *n*=5; B6 *n*=5) before ABR testing. We found that daily systemic SAHA administration did not elevate hearing thresholds compared with vehicle-treated controls (Figure 1e). These data together with our data showing systemic SAHA crosses the blood–labyrinth barrier indicate that 100 mg/kg SAHA does not negatively influence hearing function in mice.

### SAHA protects against acute ototoxicity from aminoglycoside antibiotics

**Figure 2:**
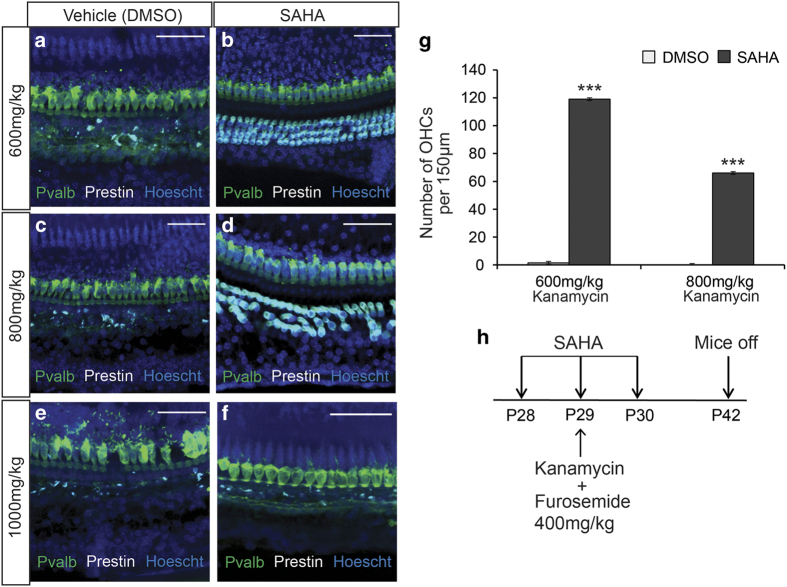
SAHA protects against kanamycin ototoxicity. Mice were administered either vehicle (DMSO) or 100 mg/kg SAHA systemically, then treated with vehicle (0.9% saline), 600 mg/kg, 800 mg/kg, or 1000 mg/kg kanamycin potentiated by 400 mg/kg furosemide. Immunofluorescence of whole-mount organ of Corti was performed using antibodies against Prestin (white), Pvalb (green), and Hoechst (blue). (**a**, **b** and **g**) Mice treated with vehicle (DMSO) and 600 mg/kg kanamycin had almost complete loss of outer hair cells, whereas SAHA-treated mice had little to no hair cell loss. (**c**, **d** and **g**) Mice receiving 800 mg/kg kanamycin and SAHA retained ~60% of the outer hair cells compared with complete loss of outer hair cells in vehicle (DMSO) control littermates. (**e**–**g**) At 1000 mg /kg kanamycin, all the mice regardless of SAHA treatment lost all their outer hair cells. (**h**) Schematic diagram depicting the dosing schedule of SAHA and kanamycin. All representative images were taken from the middle turn of the cochlea. Scale bar is 20 *μ*m.

SAHA and other HDAC inhibitors have repeatedly been shown to have neuroprotective effects *in vitro* and *in vivo* on models of inflammation, neurodegeneration, and oxidative stress.^1–3,8–10^ The formation of reactive oxygen species (ROS) and the induction of inflammatory pathways are the primary causes that underlie the molecular pathology of hair cell death related to ototoxicity.^11,12^ To determine whether SAHA protects against acute damage from ototoxicity, we used the aminoglycoside antibiotic, kanamycin, in conjunction with furosemide, a loop diuretic also known to cause ototoxicity and facilitate kanamycin crossing the blood–labyrinth barrier in mice. C57BL/6J and FVB/NJ wild-type mice received systemic administration of kanamycin by subcutaneous injection of 600 mg/kg (FVB *n*=8; B6 *n*=8), 800 mg/kg (FVB *n*=8; B6 *n*=8), or 1000 mg/kg (FVB *n*=8; B6 *n*=8) kanamycin dissolved in 0.9% saline in conjunction with intraperitoneal injection of 400 mg/kg furosemide that was given once at P29. We administered 100 mg/kg SAHA (FVB *n*=4; B6 *n*=4) or vehicle (DMSO; FVB *n*=4; B6 *n*=4) at P28 (1 day before acute damage), then at P29 (8 h after kanamycin/furosemide treatment), and at P30 (Figure 2h). It is important to note that all vehicle control mice (kanamycin vehicle, 0.9% saline and SAHA vehicle, DMSO) received 400 mg/kg furosemide; as furosemide alone (no kanamycin) combined with SAHA or vehicle had no effect (data not shown), we will only refer to whether kanamycin or SAHA/vehicle were administered. Mice were euthanized at P42, processed for immunofluorescence, and analyzed for morphologic changes to the inner ear, specifically the organ of Corti where the auditory hair cells reside. Mice treated with SAHA and 600 mg/kg kanamycin had little to no hair cell loss compared with DMSO-treated control mice, which had almost complete loss of outer hair cells (Figures 2a, b and g). Mice treated with SAHA and 800 mg/kg kanamycin retained ~60% of the outer hair cells (Figures 2c, d and g), whereas mice treated with SAHA and 1000 mg/kg kanamycin lost all outer hair cells (Figures 2e and f). These data together suggest that SAHA protects outer hair cells from kanamycin ototoxicity but very high doses of kanamycin overwhelm SAHA’s protective effect.

**Figure 3:**
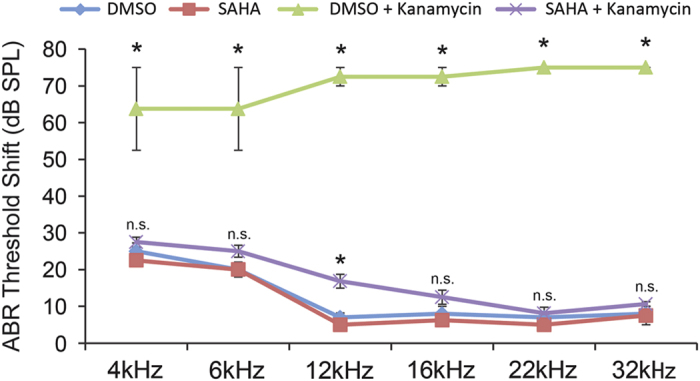
SAHA preserves hearing thresholds. Auditory brainstem responses were analyzed in mice treated with a combination of SAHA/vehicle (DMSO) and kanamycin. ABR thresholds for SAHA-treated mice that were also administered 600 mg/kg kanamycin had near-normal hearing thresholds that only differed significantly from mice that did not receive kanamycin at 12 kHz. However, vehicle (DMSO) control mice that received kanamycin had significantly elevated hearing thresholds at every frequency tested compared with vehicle (DMSO) alone, SAHA alone, or SAHA with kanamycin mice.

Although loss of outer hair cells is typically considered to be the primary cause of hearing loss from ototoxicity, hearing function relies upon the ability of the hair cells to transmit signals along the spiral ganglion, which relays the signal through the auditory portion of the vestibulocochlear nerve to the temporal lobe of the brain. To determine whether SAHA preserves hearing function, we analyzed FVB/NJ wild-type mice treated with 100 mg/kg SAHA and 600 mg/kg kanamycin for hearing thresholds at P42 by ABR (Figure 3). Using the same dosing schedule as described above (Figure 2h), mice treated with 600 mg/kg kanamycin and vehicle (DMSO) (FVB *n*=9) had significantly elevated ABR thresholds at every frequency tested compared with controls that were not administered kanamycin (FVB *n*=8). However, mice receiving both SAHA and kanamycin (FVB *n*=9) had normal hearing thresholds at every frequency tested except a small but significant threshold shift at 12 kHz compared with controls that did not receive kanamycin.

### Lack of hair cell loss is not owing to hair cell regeneration

**Figure 4:**
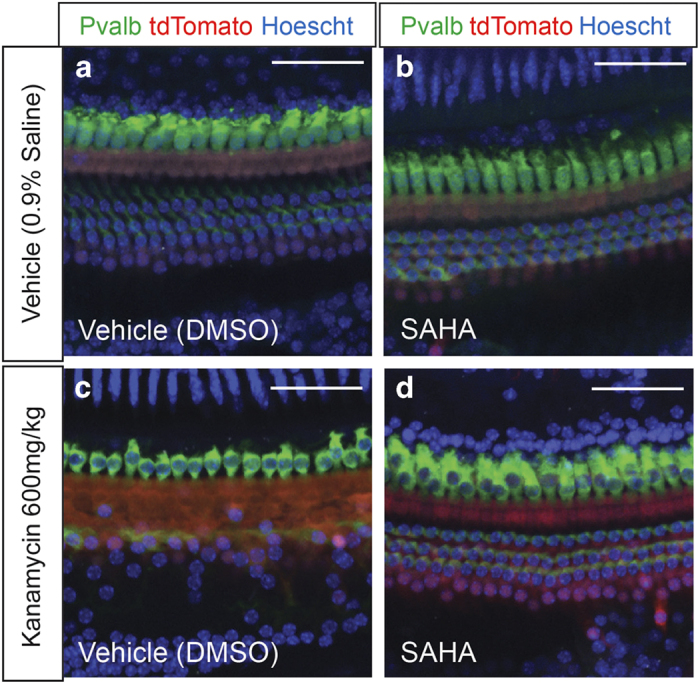
Hair cell regeneration is not facilitated by SAHA. *Fgfr3-icreER*^*T2*^; *Atoh1-HA; tdTomato* mice treated with SAHA with or without kanamycin did not generate new hair cells. (**a** and **b**) *Fgfr3-icreER*^*T2*^; *Atoh1-HA; tdTomato* mice treated with vehicle (0.9% saline) had normal-appearing outer hair cells that lack the formation of newly generated hair cells regardless of whether SAHA or vehicle (DMSO) was delivered. (**c** and **d**) Kanamycin administered to *Fgfr3-icreER*^*T2*^; *Atoh1-HA; tdTomato* mice that were treated with vehicle (DMSO) lost the vast majority of their outer hair cells, whereas SAHA-treated kanamycin mice retained their outer hair cells and did not form new hair cells. All the representative images were taken from the middle turn of the cochlea. Scale bar is 20 *μ*m.

Studies have shown that inhibitors of epigenetic events, such as DNA methylation, histone deacetylation, and histone methylation, are able to facilitate cellular reprogramming.^13–18^ Although unlikely, it is possible that SAHA may not actually be providing protection against kanamycin ototoxicity but instead SAHA may facilitate hair cell regeneration. To determine whether SAHA is able to induce hair cell regeneration, we analyzed *Fgfr3-icreER*^*T2*^*; CAG-loxP-stop-loxP-Atoh1-HA; Rosa26-CAG-loxP-stop-loxP-tdTomato* (*Fgfr3-icreER*^*T2*^; *Atoh1-HA; tdTomato)* mice for hair cell regeneration. In the auditory field, ectopic expression of the transcription factor *Atoh1* has been used to convert neonatal mammalian non-sensory cells into cells that express many endogenous hair cell markers.^19–21^ Although, ectopic expression of *Atoh1* alone can convert neonatal non-sensory cells into hair cell-like cells,^19–21^ loss of cellular plasticity at later postnatal ages prevents this conversion from occurring. At P28, *Fgfr3* is not expressed in hair cells but is highly expressed in the non-sensory supporting cells that lie beneath the outer hair cells. As supporting cells are the source of newly regenerated hair cells in non-mammalian vertebrates, the inclusion of the tdTomato reporter in our mouse model allowed us to lineage trace these cells. Intraperitoneal injection of 0.25 mg/g tamoxifen in corn oil was given to *Fgfr3-icreER*^*T2*^; *Atoh1-HA; tdTomato* mice at P28. Mice were then treated with 100 mg/kg SAHA or vehicle at P30 (one day before acute damage), then at P31 (8 h after kanamycin/furosemide treatment), and at P32. Mice received 600 mg/kg kanamycin or vehicle (0.9% saline) with 400 mg/kg furosemide at P31, euthanized at P44, then processed for immunofluorescence, and analyzed for morphology (*n*=6 for each treatment group). We were unable to detect the formation of newly generated hair cells in *Fgfr3-icreER*^*T2*^; *Atoh1-HA; tdTomato* mice that were treated with SAHA regardless of whether kanamycin was administered (Figures 4a–d). Since ectopic *Atoh1* expression in the supporting cells in conjunction with SAHA treatment should have provided the best case scenario for SAHA-mediated regeneration, we concluded that the hair cells found in our wild-type model were protected against ototoxic cell death and not newly regenerated hair cells.

### SAHA-mediated protection correlates with activation of pro-survival genes

**Figure 5:**
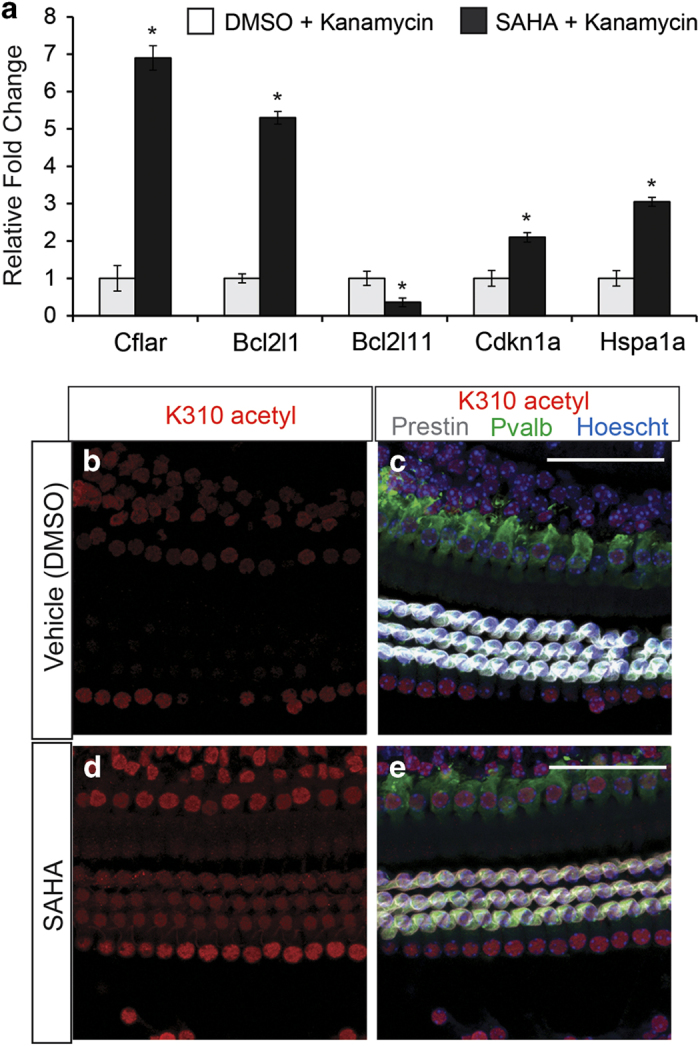
Nf-*κ*B transcription factor RelA retains K310 acetylation resulting in increased expression of pro-survival genes. Taqman gene expression assays were done on microdissected organ of Corti from animals treated with a combination of SAHA/vehicle (DMSO) and kanamycin. Expression levels are relative to endogenous gene controls *18S*, *GAPDH*, and *Actb*. Fold changes are shown relative to vehicle (DMSO)+kanamycin controls. (**a**) The expression levels of pro-survival genes *Cflar, Bcl2l1*, *Cdkn1a*, and *Hspa1a* were all increased significantly in SAHA+kanamycin-treated mice but the pro-apoptosis gene *Bcl2l11* was significantly decreased compared with vehicle (DMSO) controls. Immunofluorescence of whole-mount organ of Corti was performed using antibodies against RelA/p65 K310 acetylation (red), Prestin (white), Pvalb (green), and Hoechst (blue). (**b**–**e**) We found that mice treated with SAHA and kanamycin retained RelA/65 K310 acetyl in the nucleus of the outer hair cells but mice treated with vehicle (DMSO) and kanamycin had a complete absence of RelA/65 K310 acetyl in the nucleus. All the representative images were taken from the middle turn of the cochlea. Scale bar is 20 *μ*m.

Multiple HDAC inhibitor studies have identified components regulated by HDACs of various anti-apoptotic pathways that underlie the neuroprotective effects found in models of inflammation, neurodegeneration, and oxidative stress.^1–3,8–10^ We used the information garnered from these HDAC inhibitor studies, and combined these data with what is known about the mechanism underlying ototoxic hair cell death to identify a potential mechanism of protection in the inner ear. HDAC inhibitors have primarily been thought to regulate histone acetylation and thus affect gene expression. However, HDACs have also been shown to regulate the acetylation of non-histone proteins including Forkhead Box O (FoxO) transcription factors, Sp1, and RelA/p65, and heat shock protein 90.^4,5,22–24^ Interestingly, a recent report found that during oxidative stress Foxo3a is regulated by HDAC2, which causes Foxo3a to switch from upregulating pro-survival factor Cdkn1a/p21^CIP1^ to upregulating the pro-apoptotic factor Bcl-2-like protein 11/Bim.^23^ During oxidative stress, HDAC inhibitor treatment blocks HDAC1-mediated deacetylation of Sp1 leading to hyperacetylation, which increases Sp1 DNA binding affinity causing an upregulation of pro-survival factor Cdkn1a/p21^CIP1^.^3^ Foxo3a and Sp1 have several common target genes that are co-regulated during cellular stress,^25–27^ and each of these transcription factors has been shown to switch between pro-apoptosis to pro-survival upon HDAC inhibitor treatment by upregulating pro-survival genes such as *Cdkn1a/p21*^*CIP1*^.^3,23,27^ To test whether Foxo3a- and Sp1-regulated genes are altered following SAHA and kanamycin treatment, we measured the expression levels of *Cdkn1a/p21*^*CIP1*^, *Bcl2l11/Bim*, and *Hspa1a/Hsp70* by qPCR using Taqman probes on mRNA isolated from microdissected organ of Corti from wild-type mice using 100 mg/kg SAHA (FVB *n*=8) and 600 mg/kg kanamycin (FVB *n*=8) by our initial dosing schedule as described above (Figure 2h), but mice were euthanized 6 h after the last SAHA dose at P30. Our data show that the expression of both *Cdkn1a/p21*^*CIP1*^ and *Hspa1a/Hsp70* are significantly increased in SAHA-treated mice compared with vehicle (DMSO) controls (Figure 5a). We also found a significant decrease in the expression of the pro-apoptosis factor *Bcl2l11/Bim* in SAHA-treated mice compared with vehicle (DMSO) controls (Figure 5a), suggesting a transcriptional switch in expression from pro-apoptosis to pro-survival genes.

### SAHA facilitates the activation of the Nf-*κ*B signaling pathway

Ototoxicity studies in the mammalian inner ear have identified components of the Nf-*κ*B pro-survival pathway that are misregulated in outer hair cells leading to cell death.^11,12,28^ The Nf-*κ*B pro-survival subunit, RelA/p65, is regulated by acetylation/deacetylation of specific lysine residues. The acetylation status of RelA/p65 lysine residue K310 mediated by p300/CBP (acetylation) and HDAC3 (deacetylation) regulates RelA/p65 function both *in vivo* and *in vitro*.^29–31^ The acetylation of RelA/p65 K310 is essential for its full transcriptional activity,^29–31^ as deacetylation at K310 enhances I*κ*Bα binding and leads to I*κ*Bα-dependent nuclear export of the NF-*κ*B complex.^31^ We examined whether SAHA treatment led to the retention of RelA/p65 K310 acetylation in hair cells by immunofluorescence using antibodies specific for RelA/p65 K310 acetylation (K310acetyl). Using the 100 mg/kg SAHA and 600 mg/kg kanamycin dosing schedule as described above (Figure 2h), wild-type mice were euthanized 6 h after the last SAHA dose at P30, then processed for immunofluorescence. Similar to previous work,^28^ we found that Nf-*κ*B subunit RelA/p65 is absent from outer hair cell nuclei following aminoglycoside antibiotic treatment but present in the nuclei of all other cell types in the organ of Corti. However, our data revealed the presence of RelA/p65 K310acetyl in the nucleus of hair cells only in SAHA-treated mice (FVB *n*=8) compared with vehicle (DMSO) controls (FVB *n*=8), suggesting activation of the pro-survival Nf-*κ*B pathway (Figures 5b–e). To confirm SAHA-mediated activation of the Nf-*κ*B pathway, we measured the expression levels of known RelA/p65 target genes *Cflar/cFLIP*_*L*_ and *Bcl2l1/Bcl-xL* by qPCR using Taqman probes on mRNA isolated from microdissected organ of Corti at P30 as described above (FVB *n*=8*;* for each treatment group). SAHA-treated mice had significant increases in the expression of both *Cflar/cFLIP*_*L*_ and *Bcl2l1/Bcl-xL* compared with vehicle (DMSO) controls (Figure 5a). These data, taken together, indicate that SAHA facilitates outer hair cell survival following acute ototoxic insult by inhibiting the deacetylation of RelA/p65 K310 leading to activation of the Nf-*κ*B pro-survival pathway.

## Discussion

Here, we show for the first time that systemic delivery of the FDA-approved HDAC inhibitor, SAHA, is able to cross the mouse blood–labyrinth barrier, induce changes in histone acetylation levels, and does not negatively impact hearing function. We found that systemic SAHA treatment in mice protects against acute ototoxic insult from a combination of kanamycin and furosemide and almost completely preserves hearing thresholds. Our data show that HDAC inhibition does not facilitate hair cell regeneration, even in combination with a mouse model that ectopically expresses the hair cell differentiation factor, *Atoh1*. Our data provide evidence that SAHA protection is correlated with an increase in the expression of pro-survival genes, *Cdkn1a/p21*^*CIP1*^ and *Hspa1a/Hsp70*, and decreased expression of pro-apoptosis gene, *Bcl2l11/Bim.* In addition, we found that SAHA treatment led to RelA/p65 K310 acetylation and activation of the Nf-*κ*B pro-survival pathway with increased expression of *Cflar/cFLIP*_*L*_ and *Bcl2l1/Bcl-xL*. Altogether, these data provide strong evidence that HDAC inhibition using an FDA-approved drug protects hearing from acute ototoxic insult by blocking the deacetylation of transcription factors required for cell survival.

Previous studies have shown that aminoglycoside antibiotics cause increased histone deacetylation in mammalian hair cells through recruitment of HDACs to the chromatin.^32,33^ Although HDAC inhibitors were shown to have a protective effect on hair cells subjected to aminoglycoside antibiotics in neonatal (P3) explant cultures *in vitro*,^32^ more recent work has shown HDAC expression in the organ of Corti to be highly variable during the first postnatal week of development.^7^ In addition, mice do not develop the ability to hear until approximately P12 and the organ of Corti is not fully mature until after P15.^34^ In our model, we systemically administer SAHA to young mice with fully mature hearing before a time point associated with age-related hearing loss.

*In vivo* studies of hair cell regeneration found that both transient and irreversible induction of ectopic *Atoh1* in supporting cells during the first postnatal week, leads to the formation of hair cell-like cells.^35,36^ However, by P30, induction of *Atoh1* no longer leads to the formation of hair cell-like cells.^35,36^ These data suggest that cochlear supporting cells lose their cellular plasticity and capacity for cellular reprogramming during inner ear maturation. Studies outside the hearing field have shown that inhibitors of epigenetic events such as histone deacetylation are able to improve reprogramming efficiency.^13,14,16–18^ However, HDAC inhibition in the inner ear combined with ectopic Atoh1 was unable to facilitate cellular reprogramming. These data together indicate that hair cell lineage-specific genes in the mammalian supporting cells are tightly regulated by more permanent epigenetic marks such as histone methylation and DNA methylation. Future reprogramming studies in the inner ear will need to focus on overcoming these stable forms of epigenetic regulation.

Similar to our results, previous studies of aminoglycoside antibiotic ototoxicity have shown that only outer hair cells in the organ of Corti misregulate the pro-survival Nf-*κ*B transcription factor complex (p50+RelA/p65).^28,33^ Our data indicate that Nf-*κ*B misregulation in hair cells in response to aminoglycoside antibiotic ototoxicity is caused by HDAC-mediated deacetylation of RelA/p65 at K310 resulting in RelA/p65 nuclear exclusion and degradation. Systemic SAHA treatment inhibits the HDAC-mediated RelA/p65 K310 deacetylation leading to activation of the Nf-*κ*B pro-survival pathway and increased expression of genes involved cell survival.

Although RelA/p65 K310 deacetylation is directly mediated by HDAC3,^29–31^ HDAC1 and HDAC2 also regulate RelA/p65 access to target genes. In addition, Nf-*κ*B target genes activated by RelA/p65 have also been shown to be co-activated by Sp1.^37^ As Sp1 deacetylation is mediated by HDAC1 and Foxo3a function is regulated by HDAC2, it is likely that hair cell survival is dependent upon inhibition of multiple HDACs. Unfortunately, the hearing field continues to lack an appropriate culture system to study the mature organ of Corti, which severely limits our ability to tease out the precise molecular mechanism leading to hair cell survival. However, our data provide evidence that class I HDACs control the transcriptional activation of pro-survival pathways in response to ototoxic insult by regulating the acetylation status of transcription factors found at the crossroads of cell death and survival in the mammalian inner ear.

## Materials and Methods

### Mice

FVB/NJ and C57BL/6J wild-type mice were obtained from the Jackson Laboratory (Bar Harbor, ME, USA). *CAG-loxP-stop-loxP-Atoh1-HA*, *Fgfr3-icreER*^*T2*^, and *Rosa26-CAG-loxP-stop-loxP-tdTomato* mice were generated as previously described.^36,38,39^ Mice are housed with a 12/12 h dark/light cycle and fed *ad libitum*. Each litter was divided equally by sex and genotype into experimental and vehicle control groups. All the procedures were approved by St. Jude Children’s Research Hospital Animal Care and Use Committee (ACUC).

### Drug preparation and administration

Suberoylanilide hydroxamic acid (SAHA) was obtained from Selleck Chemical (Houston, TX, USA). SAHA was dissolved in DMSO at 50 mg/ml to be delivered in a volume that would bypass the potential detrimental effects of excessive DMSO, as described previously.^40^ Kanamycin (Sigma, St. Louis, MO, USA) was dissolved in 0.9% saline (Hospira, Lake Forest, IL, USA) and delivered by subcutaneous injection. Furosemide (Hospira) was administered by intraperitoneal injection. Tamoxifen (Sigma) was dissolved in corn oil at 25 mg/ml and delivered by intraperitoneal injection at 0.25 mg/g.

### Immunofluorescence

FVB/NJ and C57BL/6J wild-type mice and *Fgfr3-icreER*^*T2*^; *Atoh1-HA; tdTomato* mice were anesthetized with 250 mg/kg body weight tribromoethanol and perfusion fixed with 4% paraformaldehyde. Ears were removed and placed in 4% paraformaldehyde overnight at 4 °C, then incubated in 100 mM EDTA for 2 days. All the ears used for cyrosections were treated with 30% sucrose overnight at 4 °C, then flash frozen in TFM freezing medium (Triangle Biomedical Sciences, Durham, NC, USA) for cryosectioning at 12 μm. Whole-mount samples of the organ of Corti were microdissected from the surrounding inner ear tissue then placed in PBS. Following processing, samples were processed for immunofluorescence with antibodies against Histone H4ac (pan-acetylation, 1 : 1000; Active Motiff, Carlsbad, CA, USA), Pvalb (1 : 500; Sigma), Prestin (N20) (1 : 500; Santa Cruz, Dallas, TX, USA), and NF-*κ*B RelA/p65 K310 acetylation (1 : 500; Abcam, Cambridge, MA, USA). Secondary antibodies were used at 1 : 200 and conjugated with Alexa 488, Alexa 568, Alexa 647 (Invitrogen, Carlsbad, CA, USA), or HRP and then labeled with Tyramide Signal Amplification Kit (Vector Laboratories, Burlington, CA, USA). Images were captured on a confocal microscope (Zeiss LSM 700 confocal microscope (Carl Zeiss, Jena, Germany)) using the Zen 2011 software then processed in LSM Image Browser and Photoshop CS3. Laser intensity was kept consistent between samples.

### Auditory brainstem response (ABR)

Mice were anesthetized with Avertin (0.6 mg/g bodyweight, intraperitoneal) and kept on a heating pad at 37 °C. Auditory brainstem responses (ABR) were measured using a Tucker Davis Technology (TDT, Alachua, FL, USA) System III with RX6 Multiprocessors and BioSigRP software. Tone signals were calibrated using a ¼-in ACO microphone (7017, ACO Pacific, Belmont, CA, USA) connected to the RX6 multiprocessor analog/digital input. Calibrated sinusoidal signals (0.5 ms rise-time, 5 ms duration) were fed into an EC-1 electrostatic speaker using the digital/analog output of an RX6 multiprocessor (200 kHz sampling rate) and attenuated using a PA5 programmable attenuator. The speaker was connected to a plastic probe that was inserted into the mouse ear canal and placed close to the eardrum (~1 *μ*m). Frequencies tested were 4, 6, 12, 16, 22, 32, and 44 kHz with sound pressure level (dB SPL, relative to 20 *μ*Pa) attenuated in 5 dB steps between 75 and 0 dB SPL. ABR waveforms were recorded using subdermal needles placed at the vertex of the skull, below the pinna, and at the base of the tail. The needles were connected to a low-impedance head-stage (RA4LI, TDT) and fed into the optical port of the RX6 multiprocessor through a pre-amplifier (RA4PA, Gain 20x, TDT). ABR waveforms were averages obtained from 500 presentations of the tone (21/s) in alternating phase and were band-pass filtered (300–3 kHz). Thresholds in the ABR tuning curves were defined as the minimum dB SPL of a tone that elicited a wave I above the noise floor. All the experiments were conducted in a sound booth (Industrial Acoustic Company, IAC, Model 120A double wall). Two-way ANOVA followed by Student’s *t*-test with Bonferroni correction was used to compare ABR thresholds between experimental and control mice.

### RNA isolation and real-time PCR

Mice were euthanized by CO_2_ inhalation, then decapitated, ears removed by gross dissection, placed in ice-cold HBSS, and then microdissected for the organ of Corti. Organ of Corti RNA was isolated using the RNAqueous-Micro RNA Isolation Kit (Ambion, Austin, TX, USA). Isolated RNA was treated with DNase I before cDNA synthesis. cDNA was generated using Superscript First-Strand cDNA Synthesis system for RT-PCR (Invitrogen) with random primers.

Relative expression levels were assayed utilizing TaqMan Gene Expression Master Mix and TaqMan probes (Applied Biosystems, Foster City, CA, USA) for *Cflar, Bcl2l1*, *Bcl2l11*, *Cdkn1a*, *Hspa1a*, 1*8S*, *GAPDH*, and *Actb*. Reactions were run in triplicate in an Eppendorf Realplex^2^ Mastercycler System (Hauppauge, NY, USA). The level of *18S, GAPDH*, and *Actb* were used as internal controls and were run as a multiplex reaction with each assayed gene. The difference in C_T_ between the assayed gene and *18S, GAPDH*, and *Actb* for any given sample was defined as ΔC_T(X)._ The difference in ΔC_T(x)_ between two samples was defined as ΔΔC_T(X)_, which represents a relative difference in expression of the assayed gene. The fold change of the assayed gene relative to *18S, GAPDH*, and *Actb* was defined as 2^−ΔΔCT^.^41^ DataAssist software (Applied Biosystems) was used for statistical analysis and to confirm ΔC_T(X)_ calculation.

## Abbreviations

HDAC: histone deacetylase
SAHA: suberoylanilide hydroxamic acid
NSAIDS: nonsteroidal anti-inflammatory drugs
ABR: auditory brainstem response
DMSO: dimethyl sulfoxide
ROS: reactive oxygen species
FoxO: Forkhead Box O
FDA: Food and Drug Administration.
